# Adolescents’ Perceptions of Harmfulness of Tobacco and Tobacco-like Products in Finland

**DOI:** 10.3390/ijerph19031485

**Published:** 2022-01-28

**Authors:** Salma El-Amin, Jaana M. Kinnunen, Arja Rimpelä

**Affiliations:** 1 Unit of Health Sciences, Faculty of Social Sciences, Tampere University, 33014 Tampere, Finland; salma.elamin@tuni.fi (S.E.-A.); arja.rimpela@tuni.fi (A.R.); 2 Department of Adolescent Psychiatry, Tampere University Hospital, P.O. Box 2000, 33521 Tampere, Finland

**Keywords:** adolescents, perceptions, harmfulness, smoking, electronic cigarettes, snus, water pipe, nicotine

## Abstract

During the recent years, new tobacco and tobacco-like products, e.g., e-cigarettes, have emerged on the market. Adolescents often underestimate health risks in general, including those concerning tobacco. Little is known of adolescents’ perceptions of health risks of the newer products. Our paper compares adolescents’ perceptions of harmfulness of cigarettes, e-cigarettes, snus, water pipes, and nicotine in Finland, a country with a long history of strict tobacco control policy. Online surveys to nationally representative samples of 12–18-year-olds were conducted in 2017 and 2019, with 7578 answering the surveys. Only 3% of boys and 2% of girls did not agree that cigarettes are harmful to health. The percentages were slightly higher for snus (6% and 3%, respectively) and nicotine (12%, 8%) but much higher for e-cigarettes (30%, 22%) and water pipes (36%, 38%). Those who used the product, whose parents were smokers or had lower education, and whose school performance was lower, less often agreed with the harmful health effects of the products. Our results showed that adolescents understood the harmfulness of older tobacco products better than the harmfulness of the newer ones. Our results also showed the need to strengthen health education and fix adolescents’ misperceptions of the health effects of the newer products.

## 1. Introduction

During recent years, new tobacco and tobacco-like products, such as e-cigarettes, have emerged to the market. False information on their health effects is common, e.g., in social media which is widely used by adolescents. Adolescents often believe that they are invulnerable to harm caused by tobacco products and, therefore, they may underestimate the magnitude of their health risks [1,2,3]. Harmfulness perceptions may vary between the products, e.g., in some studies, e-cigarettes are perceived as less hazardous than conventional cigarettes, and particularly so among smokers [3,4]. Finland has a ban on selling tobacco and tobacco-like products to minors, a ban on all advertising including e-cigarettes, a ban on smoking and vaping in public places, in schools and restaurants and, similar to the other European Union countries, a ban on selling snus. However, Finnish adolescents are familiar with snus because it is transported from Sweden, a neighboring country, through illegal channels and with passengers who are allowed to transport a certain amount per day.

Tobacco leaves contain thousands of harmful constituents; almost all these products are known to be hazardous to health, including carcinogenic compounds and other toxins and constituents, in addition to other substances added by the manufacturers [5]. Snus is noted for its harm to health and its addictive nature [6,7,8]. A special feature of water pipe use is that even a moderate user may be exposed to a higher amount of smoke, carbon monoxide, and nicotine than a person smoking cigarettes [9]. Additionally, water pipe use is increasing in Europe [10]. Tobacco products and many tobacco-like products contain nicotine; high amounts may lead to dependence and neurobiological changes in an adolescent’s developing brain [11,12]. The use of e-cigarettes among adolescents has been a growing fashionable novelty that has become a public health concern with its increasing popularity. There is an increasing amount of evidence on the harmful health effects of e-cigarettes [13]. Using e-cigarettes might also be a start for subsequent smoking among non-smokers [14,15] and a way to end up as a dual or poly user [16].

Parents’ role in the development of their children’s perceptions on tobacco products and their harmfulness is undoubtedly important. Parents’ positive behavior towards tobacco products creates a kind of positive sentiment feeling [17] and therefore, it may contribute to adolescents’ misperceptions that tobacco and tobacco-like products are not harmful. Parents also act as role models in transferring their smoking behavior to their children [18].

Previous research indicates that smoking among adolescents varies by socio-economic status [19] which may be more complex with newer products compared to conventional smoking. However, this kind of literature is still scarce [3]. There have been variations in smokers’ knowledge about the harmful effects of cigarettes, and this variation reflects social inequalities in smoking [20]. Few studies so far have examined the influence of socio-economic status in the context of the perception of the harmfulness of tobacco-related products [21].

Poor school performance has been found to be strongly associated with adolescent smoking [22]. Previous research has also shown that low school performance is related to use of e-cigarettes [23,24]. A longitudinal study by Minkkinen et al. [25] found that low schoolwork engagement and schoolwork difficulties were risks for smoking, and poor school performance mediated this association among Finnish adolescents. We can expect that misperceptions of health effects follow the same pattern.

There are voluminous studies on tobacco use among adolescents but relatively little data on adolescents’ perceptions of the harmful effects of different tobacco and tobacco-like products in the same study which would make comparison of the different products possible. The comparison of adolescents’ perceptions would help in planning the content of health education in schools and on social media, or elsewhere where young people spend their time. Even though the knowledge on health consequences alone is not enough to influence adolescents’ behavior of substance use [26], it is an important argument and a basis for health education.

The aim of this study is to assess adolescents’ perceptions of the harmful health effects of four various tobacco and tobacco-related products—namely cigarettes, e-cigarettes, water pipe, and snus—and nicotine as an addictive constituent in Finland, a country with a long history of strict tobacco control policy. In addition, we explore whether the perceptions are related to adolescents’ own use of these products, their school performance, parents’ smoking and socio-economic status.

## 2. Materials and Methods

### 2.1. Study Procedure

The data used in this cross-sectional study originated from the Adolescent Health and Lifestyle Survey performed in 2017 and 2019, utilizing postal and online questionnaires. The questionnaires were in national languages, Finnish and Swedish. The nationally representative samples were obtained from the Finnish Population Registry Centre. Except for age and gender, that were obtained from the Registry, all other information was reported by the adolescents. The samples included all Finns aged 12, 14, 16 and 18 years old and born on selected dates in June, July, and August. The birthday sampling was used to minimize the age variation within each age group. The number of the respondents was 4058 in 2017 (response rate 43%) and 3520 in 2019 (response rate 37%). Ethics Committee of the Tampere Region approved the study protocol for both survey years. Filling in the questionnaire was considered as adolescent’s consent to participate. This was explained on the cover page of the questionnaire. No parental consent was needed according to the Ethics Committee.

### 2.2. Measures

The perception on the harmfulness of the different products was assessed with a question: “In your opinion, is the use of the following products harmful to health?” The products were: 1. Cigarettes 2. Electronic cigarettes 3. Water pipe 4. Snus. In 2019, a new item was added: 5. Nicotine. The answering options were “Yes”, “Hard to say” and “No”. In the analyses, the variables were dichotomized as “Yes” and “No” by combining “No” and “Hard to say” options into “No”. We considered “Yes” as an agreement that the adolescent perceived the mentioned product as harmful to health. “No” and “Hard to say” were combined as “No” due to the small size of the “Hard to say” category. We wanted to separate an explicit group who are convinced of health effects from those who are not convinced. For health education programs, this separation is relevant. The variables were coded as binary (“Yes” = 1, “No” = 2) for the logistic regression analysis.

Adolescents’ experience with the products was defined for each product separately by asking whether the participant had ever tried the product (cigarette, e-cigarette, water pipe, snus). The answers were dichotomized into “No” and “Yes”. The “No” groups were those who had never tried the product in question.

Adolescents’ school performance was based on the respondents’ self-assessment of her/his latest school report compared to the average level in her/his class. The answering options were “Much better”, “Slightly better”, “About the class average”, “Poorer” and “Much poorer”. The five categories were combined into three categories: “Good school performance”, “Average school performance”, and “Poor school performance”.

Parents’ smoking was asked separately for father and mother. The variable was categorized as “Neither of them smokes”, “One of them smokes”, and “Both of them smoke”.

Parents’ education was asked separately for father and mother with a question “What is your parents’ education (mark the highest level of education)?” The educational levels were combined and categorized according to the highest educational level of either parent with “High level”, “Moderate level”, and “Low level” of education. The high level was college or university degree, and low level meant only basic education.

Parents’ work situations were measured separately for father and mother with the question “What is your mother’s/father’s work situation at present?”. The answers were combined and categorized into “Both working”, “One of them working”, “Neither of them working”.

### 2.3. Data Analyses

First, the differences in the use of each tobacco and tobacco-like products between years 2017 and 2019 were analyzed. No significant differences were found. Therefore, data from the two years were merged leading to the total number of 7578 participants. Descriptive statistics were then calculated. Logistic regression models were conducted to examine the associations between adolescents’ perceptions of the harmfulness of each product, and adolescents’ experience with the product, school performance, parents’ smoking, and parental education and work situation. A crude model was first analyzed, testing the unadjusted associations for adolescents’ perceptions of harmfulness separately for each product. Then, gender and age-adjusted analyses were performed. Age and gender were adjusted for as girls were overrepresented among the respondents (57.8%) and 12-year-olds were underrepresented as their original sample was smaller compared with the other age groups (16.3% of the respondents). Last, all variables were added into the model, namely age, gender, parental highest education, parents’ work situation, school performance, and adolescents’ experimentation with the tobacco product in the analysis. The results of the analyses are presented as Odds Ratios (ORs) and 95% Confidence Intervals (95% CIs). All analyses were performed using IBM SPSS software version 25 (IBM Corp., Armonk, NY, USA).

## 3. Results

The overall prevalence for trying cigarettes was 29.8%, for e-cigarettes 25.4%, for water pipe 9.4%, and 17.7% for snus (Table 1). As summarized in Table 2, the percentages of those who reported that the tobacco product in the question is not harmful to health varied according to the product. Only very few considered cigarettes and snus as not harmful or hard to say. Concerning nicotine, the percentages were somewhat higher, especially among the boys and in older age groups. Concerning e-cigarettes, nearly a third considered them as not harmful and for water pipe, the proportion was even higher. The numbers also varied by age and gender so that for e-cigarettes and water pipe, older age groups more often than younger age groups considered them as not harmful.

Table 3 shows the ORs for the disagreement with the products’ harmfulness to health. Almost all variables were significantly associated with the disagreement of the harmfulness. Experience with the product, poor school performance, parents’ smoking, parental lower level of education, and having only one parent working were associated with the disagreement for all products. Additionally, neither of the parents working raised the odds for disagreement of the harmfulness of cigarettes and snus. For cigarettes, the association for experimenting with them was not statistically significant nor was the parents’ working status for nicotine, but the direction was the same as for the other variables.

Table 4 presents ORs for the disagreement with the products’ harmfulness to health, when all variables are adjusted for. Compared to the ORs presented in Table 3, parents’ education became insignificant for all products, while parents’ smoking and school performance remained significant for all other products, except nicotine. Having tried the studied tobacco product was related to the perception of the harmfulness, as in Table 3. The perception of harmfulness of nicotine differed from the other products as it was associated significantly only to having tried cigarettes (Table 4).

## 4. Discussion

Our study showed that Finnish adolescents know very well that cigarettes, snus, and nicotine are harmful to health. However, concerning e-cigarettes, nearly a third was not convinced about the harmfulness of the product. Concerning the water pipe, this proportion was even higher. Disagreement on the health consequences was more common among those adolescents who had tried the tobacco products, whose school performance was lower than average and whose parents smoked, had lower education, or were not working.

Our results indicate that perceptions of negative health consequences are much less often related to e-cigarettes and water pipes than to conventional cigarettes. This is in line with earlier studies [3,21]. It has also been shown that adolescents who have used tobacco products do not perceive them as harmful to health as often as the never users [3]. A study by Wang in 2019 [21] and Roditis in 2016 [3] showed that adolescents rated the risk of conventional cigarettes as highest, higher than the risks of water pipes, e-cigarettes, and smokeless products. A similar result was found among the US youth where 73% perceived e-cigarettes as less harmful than cigarettes, and only 20% of adolescents perceived smokeless tobacco harmful [27]. In our study, there was hardly any difference in the perception of harmfulness to health between snus and cigarettes. We did not ask about intentions to smoke, but Halpern-Felsher et al. [1] showed that even those who report intentions to smoke in the future estimate their chances of experiencing smoking-related negative health consequences as less likely than do non-smokers and non-intenders.

Information on the health consequences and environmental effects of cigarettes, and of passive smoking has been delivered in schools’ health education lessons, which are mandatory in Finland. The health consequences of cigarettes are also discussed continuously in the media. Therefore, it is not a surprise that the Finnish adolescents’ knowledge on the harmfulness of smoking and nicotine is at a high level. However, it is likely that education on the health consequences of e-cigarettes and water pipe has been covered less, as they are more recent products in the Finnish tobacco and nicotine market. On the other hand, the sales ban to minors in Finland includes all tobacco products and tobacco substitutes such as e-cigarettes, which could have given an impression of similar health effects. Additionally, legal restrictions, meaning a total ban, for vaping in public places, restaurants or schools are the same as those for smoking with the introduction of new national regulations [28].

Young people are active on social media and skillful in using their smart phones. So, they are likely to be misled by false information about these products presented in social media [29]. For instance, e-cigarettes in social media cover a whole range of opinions and information on various reliable and less reliable platforms. Some studies identified conflicting perceptions among social media users, and the discussion in media has been more contradictory [29]. A systematic review by Kwon and Park in 2020 [30] studied the perceptions and sentiments associated with e-cigarettes in social media and concluded that there were more positive sentiments expressed than negative ones. A study by Myslín et al. in 2013 [31] with a focus on water pipe and e-cigarettes showed similar results; sentiments towards these products were more positive overall. It is likely that social media is one of the factors which has modified adolescents’ perceptions of e-cigarettes’ health effects and the same may be true for the water pipe. In addition to social media, higher awareness about the negative health consequences of smoking cigarettes may be attributed to several reasons; for example, smokers must be more aware of the health warnings on the cigarette packages than non-smokers [32]. There are studies which have shown that the warning signs on cigarette packs can influence the perceptions of the risks of smoking [33]. Katz et al. [34] stated in 2018 that the warning labels increase uncertainty of perceptions which can lead to reduced effectiveness of warning labels and reduced intentions to avoid cigarette use among non-smokers.

Our results showed that parents’ smoking was associated with their children’s opinion that cigarettes and other tobacco and tobacco-like products are not harmful to health. This is an expected result as parents act as role models for their children, reflecting the familiarity with the information that tobacco is not harmful. This addresses the role of parents as both a source of knowledge and a positive example for their children by being tobacco-free themselves [18]. Restrictions at home, parental understanding of exposing their children to tobacco smoke, acting as a role model and correcting possible misinformation and potential misperceptions about tobacco risks may be a powerful tool to reduce the onset of using tobacco products and affect perceptions of the harmful effects of these products [17,35].

The results on the influence of parents’ education and socio-economic status on children’s smoking has varied, but parents’ smoking is a major influence [17,18,35]. It was not a surprise that the children from lower socioeconomic positions had somewhat more often misperceptions of health effects of tobacco and tobacco-like products in our study. Adolescents with only one working parent, often meaning a lower level of income in the family, had a higher risk of reporting that tobacco and tobacco-like products are not harmful when compared to adolescents with both parents working. The highest education level of parents was not strongly associated with their children’s misperception about the harmful effects of tobacco and tobacco-like products, as it was attenuated to non-significance when adjusting for other factors. These findings suggest that, compared to adolescents’ own tobacco use, parental socio-economic status has less influence on adolescents’ perception on harmfulness of tobacco and tobacco-like products [36].

Our study found a strong association between poor school performance and misperceptions about the harmfulness of cigarettes, e-cigarettes, water pipe, and snus. This association may be explained by the strong association between the use of tobacco products and low school performance [24,25]. However, it is also possible that adolescents with poorer school performance have not been as active in lessons and may have missed the taught health information.

The data used in this study was based on self-reporting and thus is subject to misclassification bias. However, the surveys that use self-report are generally valid and reliable (e.g., [37]). On the other hand, respondents’ own reports are the only way to increase understanding on perceptions because there are no objective means of measurement. The large sample size collected over two years decreases the possibility of chance variation. The response rates were low, and we cannot exclude the possibility that the prevalence figures do not completely correspond to the real ones. On the other hand, corresponding studies showed mainly similar results, and the results are believable considering that some products are new, and some have been on the market for many years. The strength of our study was that very few other studies have studied the perceptions of five tobacco and tobacco-like products in the same survey.

## 5. Conclusions

Adolescents’ understanding of health harms of the new nicotine and tobacco-like products is not adequate, and more so in vulnerable groups, i.e., those whose school performance is low, and whose parents smoke and have lower education. A key question here is adolescents’ health literacy, and whether they can distinguish false information and unreliable sources from reliable health information, particularly in social media. The new nicotine market with its emerging nicotine products is a challenge for health education, health care, health-related media, and corresponding institutions, making it difficult to keep up to-date-information of the health effects of these products. Putting more efforts into school health education on new and emerging nicotine products as well as on students’ health literacy in general could decrease misperceptions of different tobacco and tobacco-like products’ health effects. Parents’ role in adolescents’ misperceptions is important. Hence, the restrictions at home, and correcting information and potential misperceptions about the risks may also be a powerful tool in reducing adolescents’ use of different tobacco products. Parents may also need the right knowledge of the health effects. This information could be delivered through parents’ meetings by sending leaflets from schools or through health education campaigns targeted to the general population.

## Figures and Tables

**Table 1:** Percentage (%) of adolescents who had tried cigarettes, e-cigarettes, snus, and water pipe, by gender.

Experience with the Product	Boys, % (*n*)	Girls, % (*n*)	All, % (*n*)
Tried cigarettes	29.5 (934)	30.4 (1321)	29.8 (2255)
Tried e-cigarettes	30.4 (955)	21.8 (944)	25.4 (1899)
Tried water pipe	9.7 (303)	9.3 (402)	9.4 (705)
Tried snus	24.3 (766)	12.7 (554)	17.6 (1320)

**Table 2:** Percentage (%) of adolescents who reported that cigarettes, e-cigarettes, water pipe, snus, and nicotine^1^ are not harmful to health, by age and gender.

Product and Gender	12	14	16	18	All
*Cigarettes*					
Boys	4	3	4	2	3
Girls	2	3	2	1	2
*E-cigarettes*					
Boys	18	29	35	34	30
Girls	13	20	25	25	22
*Water pipe*					
Boys	29	35	39	40	36
Girls	28	34	41	44	38
*Snus*					
Boys	6	4	7	7	6
Girls	3	3	3	2	3
*Nicotine* ^1^					
Boys	6	11	16	15	12
Girls	9	9	8	7	8

^1^ Nicotine was asked only in 2019.

**Table 3:** Age and gender adjusted Odds Ratios (ORs) and 95% CIs for adolescents who reported that cigarettes, e-cigarettes, water pipe, snus, and nicotine are not harmful to health, by experimentation with the product and by socio-economic background.

Variables	Cigarettes	E-Cigarettes	Water Pipe	Snus	Nicotine
*Tried the product* ^1^					
No	1.00	1.00	1.00	1.00	1.00
Yes	1.34 (0.94–1.90)	2.84 (2.51–3.21)	2.13 (1.81–2.51)	2.65 (2.02–3.47)	2.48 (1.91–3.21)
*School performance*					
Good	1.00	1.00	1.00	1.00	1.00
Average	1.34 (0.97–1.84)	1.44 (1.28–1.61)	1.34 (1.21–1.48)	1.67 (1.30–2.15)	1.30 (1.02–1.67)
Poor	1.74 (1.11–2.75)	2.12 (1.78–2.52)	1.79 (1.52–2.11)	2.52 (1.80–3.53)	1.75 (1.24–2.48)
*Parental smoking*					
Neither smoke	1.00	1.00	1.00	1.00	1.00
One smokes	1.02 (0.69–1.52)	1.59 (1.39–1.81)	1.45 (1.28–1.64)	1.41 (1.06–1.86)	1.38 (1.04–1.83)
Both smoke	2.16 (1.30–3.58)	1.95 (1.56–2.43)	1.62 (1.31–2.00)	2.01 (1.33–3.06)	1.81 (1.13–2.88)
*Parental highest education*					
Higher level	1.00	1.00	1.00	1.00	1.00
Moderate level	3.09 (1.21–7.94)	1.91 (1.24–2.95)	1.36 (0.90–2.06)	1.61 (0.64–4.07)	1.63 (0.63–4.26)
Lower level	1.40 (1.00–1.94)	1.35 (1.21–1.51)	1.23 (1.11–1.36)	1.43 (1.11–1.83)	1.42 (1.11–1.80)
*Parental work situation*					
Both work	1.00	1.00	1.00	1.00	1.00
One works	1.95 (1.39–2.72)	1.40 (1.23–1.60)	1.28 (1.13–1.45)	1.50 (1.14–1.96)	1.07 (0.80–1.45)
Neither work	2.77 (1.32–5.79)	1.13 (0.78–1.62)	0.90 (0.64–1.26)	2.00 (1.07–3.76)	1.66 (0.48–5.69)

^1^ For nicotine, tried cigarettes was used.

**Table 4:** Adjusted Odds Ratios (ORs) and 95% CIs for adolescents who reported that cigarettes, e-cigarettes, water pipe, snus, and nicotine are not harmful to health, by experimentation with the product and by socio-economic background.

Variables	Cigarettes	E-Cigarettes	Water Pipe	Snus	Nicotine
*Tried the product* ^1^					
No	1.00	1.00	1.00	1.00	1.00
Yes	1.17 (0.79–1.75)	2.58 (2.26–2.95)	2.00 (1.68–2.38)	2.46 (1.83–3.31)	1.76 (1.19–2.60)
*School performance*					
Good	1.00	1.00	1.00	1.00	1.00
Average	1.21 (0.84–1.75)	1.23 (1.08–1.39)	1.24 (1.11–1.39)	1.43 (1.08–1.88)	1.05 (0.80–1.38)
Poor	1.76 (1.05–2.93)	1.59 (1.31–1.93)	1.59 (1.33–1.90)	1.92 (1.32–2.80)	1.05 (0.70–1.58)
*Parental smoking*					
Neither smoke	1.00	1.00	1.00	1.00	1.00
One smokes	0.78 (0.48–1.25)	1.29 (1.11–1.50)	1.27 (1.11–1.46)	1.18 (0.86–1.61)	1.16 (0.84–1.60)
Both smoke	1.97 (1.13–3.43)	1.50 (1.17–1.92)	1.36 (1.08–1.72)	1.67 (1.06–2.61)	1.55 (0.94–2.55)
*Parental highest education*					
High level	1.00	1.00	1.00	1.00	1.00
Moderate level	2.40 (0.90–6.36)	1.46 (0.91–2.35)	1.22 (0.78–1.92)	1.28 (0.50–3.31)	1.27 (0.43–3.72)
Low level	1.07 (0.75–1.53)	1.09 (0.96–1.23)	1.09 (0.98–1.21)	1.08 (0.83–1.41)	1.18 (0.91–1.54)
*Parental work situation*					
Both work	1.00	1.00	1.00	1.00	1.00
One works	1.72 (1.15–2.57)	1.27 (1.09–1.48)	1.19 (1.03–1.37)	1.37 (1.00–1.88)	0.98 (0.69–1.39)
Neither work	1.99 (0.70–5.69)	0.66 (0.40–1.11)	0.75 (0.48–1.16)	1.24 (0.49–3.15)	–

^1^ For nicotine, tried cigarettes was used.

## Data Availability

The data presented in this study are available on request from the authors with a research plan and a signed contract with Tampere University. The data are not yet publicly available due to it is being prepared for public archiving.
